# Engaging Families in Function-Focused Care: Goal Attainment and Associated Outcomes

**DOI:** 10.1093/geroni/igaa057.2751

**Published:** 2020-12-16

**Authors:** Marie Boltz, Joanne Roman Jones, Robin Hermann

**Affiliations:** 1 Penn State University, University Park, Pennsylvania, United States; 2 Pennsylvania State University, State College, Pennsylvania, United States; 3 Hospital of the University of Pennsylvania, Philadelphia, Pennsylvania, United States

## Abstract

Partnering with families to develop function-focused plans for hospitalized persons with dementia (PWD) improves both the hospital experience and patient outcomes. This secondary analysis included patients enrolled in the intervention arm of the on-going Family-centered Function-focused Care (Fam-FFC).study. This study examined the goals co-established by family caregivers, PWD, and nurses to prevent hospital-acquired complications and promote functional and cognitive recovery. The influence of goal attainment upon delirium and physical function at discharge was also examined. The majority of patients (N=162) were female (65%), black (53%) with a mean age of 82.7 (SD= 8.2). Goal attainment ranged from -2 to 2; mean = -0.24 (SD= 0.75). The goals (N=432) represent three main areas: mobility, self-care, and cognitive stimulation. Controlling for age and admission function, goal attainment was associated with less discharge delirium (F=3.2, p = .022) but not discharge function. Results support the contribution of function-focused care to improving delirium outcomes.

